# Modeling the Additive Effects of Nanoparticles and Polymers on Hydrogel Mechanical Properties Using Multifactor Analysis

**DOI:** 10.3390/nano12244461

**Published:** 2022-12-15

**Authors:** Emma Barrett-Catton, Kyle Pedersen, Maryam Mobed-Miremadi, Prashanth Asuri

**Affiliations:** Department of Bioengineering, Santa Clara University, Santa Clara, CA 95053, USA

**Keywords:** hydrogel nanocomposites, rheological properties, interpenetrating polymer networks, hydrogel–filler interactions, multivariate analysis, Han plot

## Abstract

Interpenetrating networks (IPN)s have been conceived as a biomimetic tool to tune hydrogel mechanical properties to the desired target formulations. In this study, the rheological behavior of acrylamide (AAm) [2.5–10%] hydrogels crosslinked with N,N′-methylenebis(acrylamide) (Bis) [0.0625–0.25%] was characterized in terms of the saturation modulus affected by the interaction of silica nanoparticle (SiNP) nanofillers [0–5%] and dextran [0–2%] at a frequency of 1 Hz and strain rate of 1% after a gelation period of 90 min. For single-network hydrogels, a prominent transition was observed at 0.125% Bis for 2.5% AAm and 0.25% Bis for 5% AAm across the SiNP concentrations and was validated by retrospective 3-level factorial design models, as characterized by deviation from linearity in the saturation region (R^2^ = 0.86). IPN hydrogels resulting from the addition of dextran to the single network in the pre-saturation region, as outlined by the strong goodness of fit (R^2^= 0.99), exhibited a correlated increase in the elastic (G’) and viscous moduli (G”). While increasing the dextran concentrations [0–2%] and MW [100 kDa and 500 kDa] regulated the increase in G’, saturation in G” or the loss tangent (tan(δ)) was not recorded within the observed operating windows. Results of multifactor analysis conducted on Han plots in terms of the elastic gains indicate that amongst the factors modulating the viscoelasticity of the IPN hydrogels, dextran concentration is the most important (R_Dex_ = 35.3 dB), followed by nanoparticle concentration (R_SiNP_ = 7.7 dB) and dextran molecular weight (R_MW_ = 2.9 dB). The results demonstrate how the Han plot may be systematically used to quantify the main effects of intensive thermodynamic properties on rheological phase transition in interpenetrating networks where traditional multifactor analyses cannot resolve statistical significance.

## 1. Introduction

Hydrogels are highly porous three-dimensional polymer networks with physical and biochemical properties that make them ideal for many biomedical applications such as tissue scaffolds, drug delivery, cell supports, and corneal implants [1,2,3,4,5]. However, unmodified hydrogels typically have low mechanical strength, which limits their applicability such as use for load bearing tissue scaffolds, for example, bone regeneration and artificial cartilage [1,3,6]. Recent studies have developed a number of ways to improve the mechanical properties of hydrogels including through the incorporation of additives such as polymers and nanoparticles [1,3,6,7,8,9,10,11,12]. The use of polymer additives, either to create interpenetrating polymer networks (IPN) in which the hydrogel is made of two different crosslinked polymers, or semi-IPNs in which only one of the polymers is crosslinked and the other polymer is trapped within this crosslinked network, is a common way to improve the mechanical properties [1,13,14,15,16,17,18,19,20]. For example, one study found that chitosan (CS)-poly(acrylic acid) (PAA) IPNs resulted in a hydrogel with higher tensile strength and adsorption capacity than either the CS or PAA hydrogels, resulting in a wider range of applications for the hydrogel [21]. Incorporating nanoparticles such as clay nanoparticles and graphene oxide nanosheets into hydrogels can also improve the mechanical properties of single network hydrogels [8,9,10,11,12]. Several studies have also demonstrated the use of nanoparticles such as cellulose nanocrystals, zinc oxide nanoparticles, and modified bentonite nanoparticles to improve the mechanical properties of IPN hydrogels [3,6,7]. Incorporating nanoparticles can also add or enhance other properties of IPN hydrogels such as improving the refractive index of the hydrogels, adding antibacterial properties, or improving absorbency [6,22,23]. 

Our lab has previously used both nanoparticles and polymer fillers to improve the mechanical properties of hydrogels, and study the interactions between silica nanoparticles and hydrogel networks. The addition of silica nanoparticles improved the mechanical and thermal properties of the hydrogels, a finding also reported by other groups [24,25,26]. These works support the hypothesis that silica nanoparticles enhance hydrogel properties by meditating pseudo crosslinking of pAAm networks. They also demonstrated that the improvements in mechanical strength for both single-network and IPN hydrogels was dependent on the nanoparticle–polymer interactions [24]. However, there appears to be an upper limit to the gains in mechanical strength that can be achieved with the addition of nanoparticles [27,28,29].

Various researchers have used statistical analysis translated into multifactorial modeling to study and optimize the combinatorial properties of hydrogels suitable for an array of biomedical applications [20,30,31,32]. A number of research groups have used factorial designs to optimize certain properties of hydrogel formulations such as swelling, drug release, and cell seeding [20,30,31,32]. For instance, Gajra et al. used a factorial design to investigate the effects of a number of factors including the number of freeze/thaw cycles and concentration of poly(vinyl alcohol) on drug release time [31]. In parallel, several researchers have used analysis of variance (ANOVA) in IPN hydrogel design in terms of attributes such as mechanical strength [33], biocompatibility [34,35,36], and electrical properties [37,38].

In this study, prior data on the incorporation of silica nanoparticles into a pAAm hydrogel (single network) was first evaluated using factorial analysis to determine the impact of the factors and their interactions on the elastic moduli of the hydrogels. Next, dextran was added to the system to create IPN hydrogel nanocomposites and offer an additional orthogonal approach to further improve the mechanical properties of the hydrogels. Dextran is a hydrophilic and nontoxic polysaccharide that has been used for several different tissue engineering and biomedical applications due to its biocompatibility [13,39,40,41]. Studies have shown that the fluidity and strength of hydrogels can be altered by the addition of dextran of various molecular weights [39,42]. For instance, pAAm-dextran hydrogels have higher responsiveness, swelling ratios, and enhanced mechanical properties compared to pAAm gels [1,4,5]. Therefore, pAAm-dextran hydrogels that incorporate silica nanoparticles were prepared and characterized to further investigate the relative and additive roles of dextran and nanoparticles on the hydrogel mechanical properties. Design of experiments was used to identify a baseline predictive region in the single network to further investigate the viscoelastic properties and the ranking of the main effects of the chemical crosslinker, silica nanoparticles, and dextran on the rheological properties of the IPN hydrogels.

## 2. Materials and Methods

### 2.1. Materials

All of the materials for the preparation of the single network and IPN hydrogels are as follows: acrylamide (AAm, 40% *w/v*), N,N′-methylenebis(acrylamide) (Bis, 2% *w/v*), ammonium persulfate (APS), N,N,N′,N′-tetramethyl ethylenediamine (TEMED), and low- and high-molecular weight dextran (100 kDa and 500 kDa) were purchased from Sigma Aldrich (St. Louis, MO, USA) and used as received. A Binzil silica nanoparticle (SiNP) colloid solution with a mean particle size of 4 nm was obtained as a gift from AkzoNobel Pulp and Performance Chemicals Inc. (Marietta, GA, USA). Tris–HCl buffer (pH 7.2) was purchased from Life Technologies (Carlsbad, CA, USA).

### 2.2. Polymerization Reaction

Single network hydrogel samples (pAAm) were prepared as previously reported [24]. Briefly, AAm and Bis stocks were diluted to their desired concentrations in pH 7.2, 250 mM Tris-HCl buffer, followed by the addition of TEMED (0.1% of the final reaction volume) and 10% *w/v* APS solution (1% of the final reaction volume). For the pAAm-dextran IPN hydrogel samples, 100 kDa and 500 kDa dextran was first dissolved in Tris–HCl buffer by stirring at room temperature, and then diluted to the desired concentrations before adding it to the reaction mixture prior to the addition of APS and TEMED. Similarly, for the nanocomposite hydrogels, various amounts of SiNPs were added to the single network or IPN hydrogel reaction mixtures prior to the addition of APS and TEMED.

### 2.3. Rheological Measurements

Rheological measurements of the single network and IPN hydrogels with or without nanoparticles were carried out using the MCR302 rotational rheometer (Anton Paar, Graz, Austria) using parallel plate geometry, as described previously [27]. Briefly, 500 μL of a well-mixed reaction mixture was pipetted onto the lower plate of the rheometer. The upper plate was lowered until the desired gap distance (1 mm) was achieved. Amplitude sweeps at a constant frequency of 1 Hz were then carried out to ensure measurements were carried out in the linear viscoelastic regime of the hydrogels. Next, dynamic sweep tests over frequencies ranging from 0.1 to 100 Hz were recorded in the linear viscoelastic regimes (strain amplitude of 0.01) to determine the shear elastic modulus. Elastic and viscous moduli were determined by following the gelation for 90 min (to ensure complete gelation; gelation usually occurs within 20 min) at 1 Hz and 1% strain for all samples. Relative elastic (G’) and viscous moduli (G”) were calculated by normalizing the values for the pAAm-SiNP hydrogels to the corresponding values for the control pAAm gels. The moduli are related by the tangent of the loss factor (delta) given by Equation (1).

(1) $$\tan\left(\delta \right)=\frac{G\prime \prime }{G\prime }$$

All measurements were carried out at room temperature and the final parameters were reported as an average of three independent measurements.

### 2.4. Factorial Design

A retrospective design of experiments was carried out for the single network hydrogels using the elastic shear modulus (G’) as a response. The effect of curvature was examined by combinations of 3 level factorial designs generated using JMP-SAS v.16 (JMP Statistical Discovery LLC, Cary, NC, USA) and conducted over the experimental ranges outlined in Table 1 and Table 2. Results were analyzed according to the methodology proposed by Box et al. [43] and subsequently validated by software. The levels of coded and uncoded variables as well as respective responses and associated hydrogel network types are presented throughout the study.

### 2.5. Multivariate Analysis of Variance

Multivariate analysis of variance (MANOVA) was conducted for the IPN hydrogels using MATLAB version r2021b with a significance threshold of 0.05 according to the variable and levels outlined in Table 2. The concentration of the acrylamide (AAm) was fixed at 5%. The responses were G’, G”, and tan(δ).

### 2.6. Discriminant Analysis Using Han Plots

Han plots were generated to discriminate between the elastic and viscous rheological behaviors of the polymer networks. This method is used to investigate the effect of intensive thermodynamic properties on rheological phase transition [44,45]. As polymer, cross-linker, and filler concentrations are also intensive properties governing the viscoelastic state, the use of the method was justified. For each plot, the Euclidean distance between the discrimination axis and datapoints, denoted as elastic gain, was calculated and averaged across levels for the IPN hydrogel design variables outlined in Table 2 and ranked in order of importance using Equations (2) and (3).

(2) $$D_{i}=10\log_{10}\left(\underset{\mathrm{ij}}{\sum }\frac{X}{L}\right)$$

Difference, *D_i_*, was given by Equation (2), where *X* is the Euclidean distance at a given level averaged over the number of levels (*L*). Rank, *R*, expressing the significance of the variable in terms of the range of difference *D_i_*, is given by Equation (3).

(3) $$R_{i}=\mathrm{Maximum}\left(D_{i}\right)-\mathrm{Minimum}\left(D_{i}\right)$$

Equations (2) and (3) will also be used to evaluate the change in elasticity in terms of G’, from the single network initial condition to the final IPN hydrogel configuration expressed by the variable ΔG’. A positive difference indicates an increase in G’ due to dextran incorporation.

## 3. Results

### 3.1. Saturation in Nanoparticle Mediated Enhancement of Hydrogel Mechanical Properties

**Figure 1:**
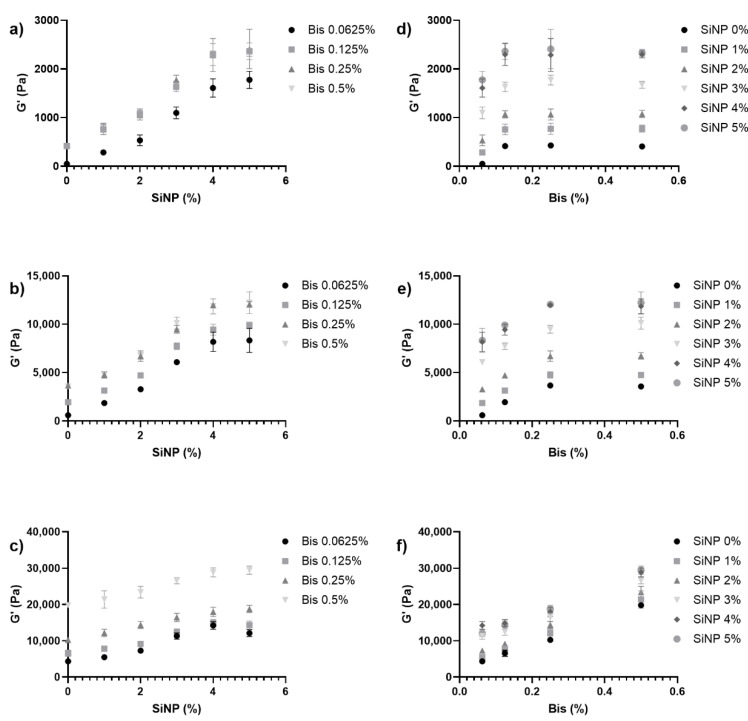
Mechanical properties of the pAAm hydrogels prepared using different concentrations of 4 nm silica nanoparticles, Bis crosslinker, and acrylamide. (**a**–**c**) Elastic moduli (G’) as a function of silica nanoparticle concentration for hydrogels prepared using different concentrations of the Bis crosslinker—●: 0.0625% Bis, ■: 0.125% Bis, ▲: 0.25% Bis, and ▼: 0.5% Bis and (**a**) 2.5% AAm, (**b**) 5% AAm, or (**c**) 10% AAm. (**d**–**f**) Elastic moduli (G’) as a function of Bis concentration for hydrogels prepared using different concentrations of nanoparticles—●: 0% SiNP, ■: 1% SiNP, ▲: 2% SiNP, ▼: 3% SiNP, ◆: 4% SiNP, and ○: 5% SiNP and (**d**) 2.5% AAm, (**e**) 5% AAm, or (**f**) 10% AAm. N = 3 for each hydrogel preparation.

First, the impact of the incorporation of nanoparticles as well as different polymer and crosslinker concentrations on the elastic modulus (G’) was investigated. Various concentrations of silica nanoparticles, AAm polymers, and Bis crosslinkers were used to create single network hydrogels, and the G’ values were investigated for various formulations of single network hydrogels. Upon preliminary investigation, the hydrogels exhibited a saturation at 4% SiNPs, which can be visualized in the plot of G’ values versus SiNP percentage (Figure 1a–c). The hydrogels also exhibited a saturation trend with respect to Bis concentration, as shown in Figure 1d–f. The saturation behaviors for the hydrogels with respect to Bis and SiNP concentrations were less pronounced for the highest AAm concentration of 10%.

### 3.2. Factorial Design Models Identify the Significance of Hydrogel Nanocomposite Parameters

To further model the curvature observed at higher nanoparticle concentrations using the design parameters, 3 level factorial designs (N = 27 runs) using either high or low Bis concentration ranges were analyzed using the values presented in Tables S1–S3 at higher SiNP values. The regression equation for the 3^3^ design is given by Equation (4). Parameters and their respective significance levels are reported in Table 3.

(4) $$\;\hat{Y}\;=\beta_{0}+\beta_{1}X_{1}+\beta_{2}X_{2}+\beta_{3}X_{3}+\beta_{12}X_{1}X_{2}+\beta_{23}X_{2}X_{3}+\beta_{13}X_{1}X_{3}+\beta_{123}X_{1}X_{2}X_{3}$$

**Figure 2:**
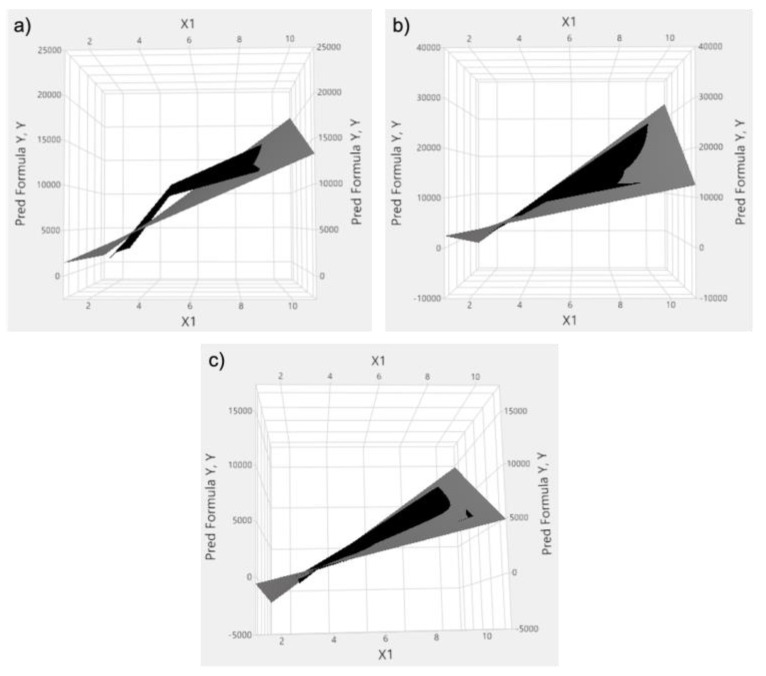
Factorial design surfaces for (**a**) pAAm hydrogels with low Bis and high silica nanoparticle concentrations, (**b**) high Bis and high silica nanoparticle concentrations, and (**c**) low Bis and low silica nanoparticle concentrations. The predicted Y surface (Pred Formula Y) modeled by Equation (4) is shown in grey to which the actual experimental surface (Y) is superimposed in black for all three models in Table 3, where X_1_ is represents the AAm concentration.

At lower Bis concentrations, the regression model was dominated by the effects of AAm (X_1_) and SiNP (X_3_) at a significance threshold of 0.1 (*p* = 0.1), as deviation from linearity is characterized by an R^2^ value of 0.86 (Figure 2a). At higher Bis concentration, the regression model was dominated by the effects of AAm (X_1_), Bis (X_2_), and their respective interaction (X_1_ × X_2_) (*p* = 0.1), characterized by an adequate coefficient of determination of 0.97. For the former, the effect of curvature could be observed in the mid-section of the surface plot due to the gap between the experimental data and predicted model (Figure 2a). For the latter, the superimposition of the empirical and theoretical was seamless, except for the lower limit region of the AAm (2.5%) (Figure 2b).

In order to operate in a region where the rheological behavior of the system is below the saturation region, as presented in Figure 2a (G’ vs. SiNP), another 3^3^ factorial design, outlined in Table S3, was conducted in the lower SiNP range. This model is dominated by the main effects (X_1_, X_2_, X_3_) and AAm/Bis (X_1_ × X_2_) as well AAm/SiNP (X_1_ × X_3_) interactions. The fact that the significance threshold has the same magnitude (*p* < 0.0001) for the main effects as well as the interactions is indicative of the onset of the competitive mechanisms for crosslinking between the Bis and SiNP. The strength of the model is reflected by an R^2^ value of 0.9912 and the superimposition of the theoretical and experimental findings in the surface response plots except for the lower limit region of the AAm (2.5%) (Figure 2c). Combining the analytical results from the models above, the operating window for the IPN hydrogels at the mid AAm level (5%) and below saturation was proposed.

### 3.3. Design of the Interpenetrating Network Hydrogel Nanocomposite Studies

Dextran was used to make IPN hydrogels to study the combined effects of dextran and SiNPs on the hydrogel elastic moduli, as previous studies have demonstrated additive effects of different fillers on hydrogel properties [1,3,6,7,8,9,10,11,12]. As described previously, one of the goals of the work was to investigate the individual and additive impacts of incorporating different fillers. It was therefore important to use SiNP values in the linear range of the single network. In a similar vein, Bis concentrations below saturation were used—in the previous study, the G’ values saturated at 0.25% Bis, so Bis concentrations at and below 0.25% were selected [29]. Dextran concentrations and molecular weights were chosen based on the literature [4]. Additionally, a single AAm concentration was selected to study the interpenetrating networks because AAm (X_1_) was a highly significant factor for the single network data throughout the three models proposed by Equation (4), as the goal was to focus more on the impact of dextran and the interactions between the variables for the IPN hydrogel studies.

### 3.4. Effect of Dextran on the Elastic and Viscous Properties of Hydrogel Nanocomposites

**Figure 3:**
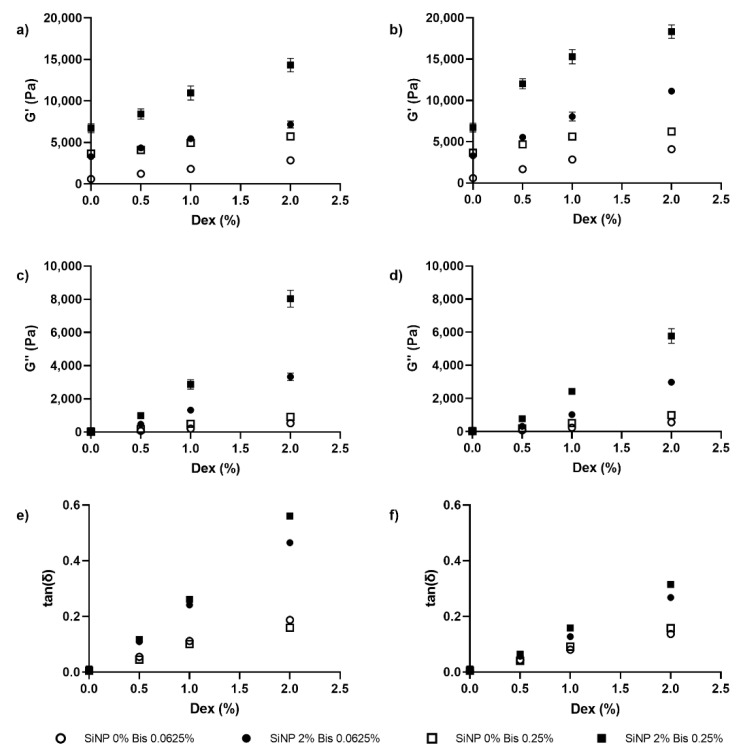
Mechanical properties of pAAm hydrogels as a function of dextran concentration prepared using different concentrations of silica nanoparticles and Bis crosslinker—○: 0% SiNP/0.0625% Bis, ●: 2% SiNP/0.0625% Bis, □: 0% SiNP/0.25% Bis, and ■: 2% SiNP/0.25% Bis, and different molecular weights of Dex—elastic moduli (G’): (**a**) 100 kDa and (**b**) 500 kDa Dex; viscous moduli (G”): (**c**) 100 kDa and (**d**) 500 kDa Dex; and loss tangent (tan(δ)): (**e**) 100 kDa and (**f**) 500 kDa Dex. N = 3 for each hydrogel preparation.

Using the parameter selection outlined in Section 3.3., pAAm-Dex-SiNP IPN hydrogel samples were prepared to investigate the role of dextran on hydrogel nanocomposites. Although there was no evidence of the saturation of G’ for dextran with a molecular weight of 100 kDa (Figure 3a), an onset of saturation was observed at 1% dextran for the higher molecular weight (500 kDa) (Figure 3b). This saturation was more prominent for hydrogel nanocomposites prepared with a higher concentration of Bis (Figure S1 and Table S4). Furthermore, the effect of dextran on G’ was more pronounced for pAAm hydrogels with SiNP (2% SiNP samples) compared to those without SiNP (0% SiNP). Collectively, the results indicate an interaction between dextran and SiNP for the IPN hydrogel nanocomposites that was more pronounced at higher Bis concentrations. 

Leveraging previous results that demonstrated the independent effect of nanoparticles and polymer fillers on hydrogel viscous modulus, this study also investigated the combined impact of these fillers on the viscous modulus of pAAm hydrogels [28,39,40]. As the dextran concentration and molecular weight increased, there was also an increase in G” (Figure 3c,d). However, in contrast with G’, there was no saturation for G”, and correspondingly for tan(δ), at higher concentrations of dextran. These results suggest that the maximum potential for fluidity of the pAAm-Dex IPN hydrogel nanocomposites has not been reached (Figure 3e,f). 

### 3.5. Statistical Analyses of the Interpenetrating Network Hydrogel Nanocomposites

Multifactor statistical methods aimed at characterizing the additive effect of dextran enabling the formation of the IPN hydrogels are summarized below. Results of the MANOVA analysis investigating the main effects in terms of the rheological parameters for the interpenetrating network are presented in Table S4. All factors were significant (*p* < 0.05) for the related rheological parameters examined, the exception being the MW of the dextran for the viscous modulus and loss factor. 

**Figure 4:**
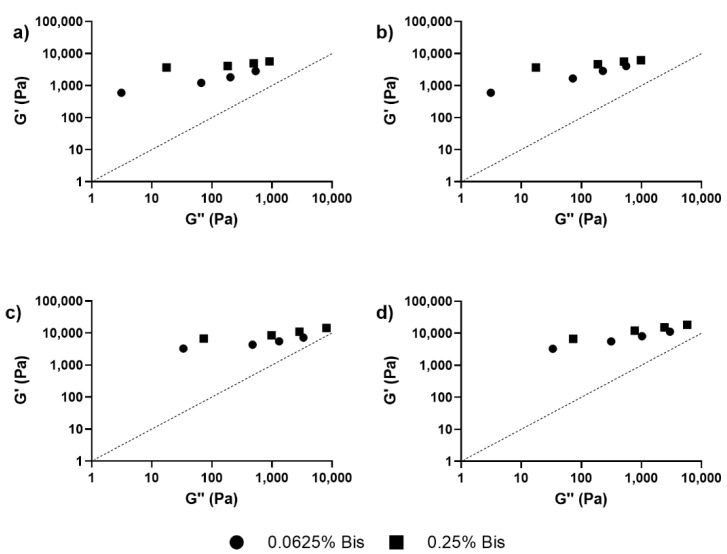
Han plots for pAAm hydrogels prepared using different concentrations of the Bis crosslinker—●: 0.0625% Bis and ■: 0.25% Bis, and different concentrations of Dex—0, 0.5, 1, and 2%, with increasing concentration going from left to right in each plot, and (**a**) 0% SiNPs and 100 kDa Dex, (**b**) 0% SiNPs and 500 kDa Dex, (**c**) 2% SiNPs and 100 kDa Dex, (**d**) 2% SiNPs and 500 kDa Dex.

As this preliminary result does not lend itself to a quantitative pareto effect, the analysis was extended to Han plots. Han plots displaying the discrimination between elastic and viscous behavior as a function of dextran concentration are presented across the SiNP, Bis, and molecular weights of dextran in Figure 4a–d. All datapoints reside over the 45° discrimination axis, indicating that the system is cross-linked. Furthermore, the data demonstrates the effect of dextran on the viscous modulus of the IPN hydrogel nanocomposites, as exhibited by the higher elastic than viscous behavior at lower dextran concentrations. 

**Figure 5:**
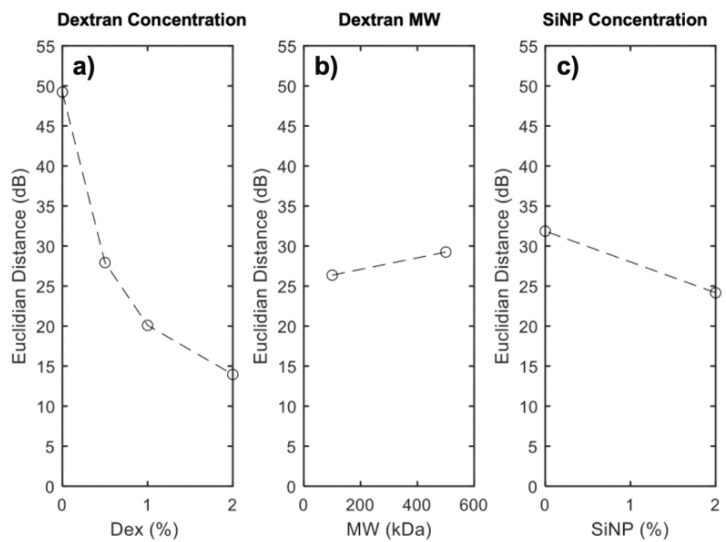
Euclidean distance from the Han plots for (**a**) dextran concentration, (**b**) dextran molecular weight, and (**c**) silica nanoparticle concentration.

Figure 5 shows the elastic gains calculated using the Euclidean distance in the Han plots. For the dextran (Figure 5a) and SiNP (Figure 5b) concentrations, the elastic gain decreased monotonously, in contrast to the effect of MW (Figure 5c). In terms of ranking the factors modulating the viscoelastic behavior, dextran concentration was the most important (R_Dex_ = 35.3 dB), followed by the effects of nanoparticles (R_SiNP_ = 7.7 dB) and the dextran molecular weight (R_MW_ = 2.9 dB).

The increase in the elastic moduli computed from the comparison of single and interpenetrating network formulations due to dextran incorporation, in terms of ΔG’ values, are presented in Table 4. In terms of ranking the factors modulating the elastic behavior, dextran concentration was the most important (R_Dex_ = 4.46 dB), followed by the effects of the dextran MW (R_MW_ = 1.53 dB) and nanoparticles (R_SiNP_ = 1.21 dB).

## 4. Discussion and Future Work

The goal of this work was to investigate the impact of incorporating two different additives on the mechanical properties of hydrogels. Other groups have previously explored combining two different additives: (a) nanoparticles to form nanocomposites, and (b) other polymers to form an IPN hydrogel to increase the mechanical strength of hydrogels [29,39]. To combine these approaches, dextran was added to the pAAm-SiNP nanocomposite hydrogels to form an IPN hydrogel nanocomposite. By combining SiNPs and dextran, the mechanical properties of the hydrogels increased beyond the saturation point for SiNPs, consistent with studies reported by other groups [24,46,47]. Results from these experiments also indicated a saturation behavior in the increases in elastic modulus for the IPN hydrogel nanocomposites, which indicates the existence of a ‘global’ saturation point, beyond the ‘local’ saturation observed for either Bis- or SiNP-mediated enhancements. Interestingly, the viscous modulus did not saturate, and, in fact, increased with increasing dextran concentrations, as confirmed by the Han plots (Figure 4). As stated in previous literature, the incorporation of additives that do not lead to chemical crosslinking into hydrogels can lead to increases in G” [29,39]. These results demonstrate that the use of different additives may allow us to independently control the elastic and viscous modulus of hydrogels. 

Statistical modeling was used to interpret the data and plan subsequent experiments. For the single network hydrogel, a factorial design was used to model the data, thereby narrowing down the experimental space for the IPN hydrogel tests, a by-product of which may be the reduction of environmental waste. First, the impact of incorporating nanoparticles (SiNPs) into pAAm hydrogels was analyzed. The G’ values for pAAm hydrogels saturated at higher concentrations of SiNPs, indicating a maximum impact of SiNPs on the elastic modulus. This saturation behavior was observed using a 3^3^ factorial design, which illustrated the curvature of the system in a 3D space, along with 2D plots of the data, which also present the curvature (Figure 2a–c). The experimental space bound by the ranges of the variables in Equation (4) highlight the saturation of G’ with increasing concentrations of Bis and Aam, and, as a result, subsequent experiments focused on low concentrations of Bis, and one concentration of AAm, to better see the impact of dextran on the hydrogels. As the use of factorial ANOVA did not discriminate between the main effects of the IPN hydrogel design matrix outlined in Table 2, the Euclidean distance to the 45° discrimination axis in the Han plot was used as an optimization parameter to rank the factors (Figure 5).

Future studies will encompass the effect of frequency on complex moduli explored through the Carreau Yasuda model used for the characterization of shear-thinning behavior of the modeled polymer nanocomposite formulations [48,49]. Frequency sweep analysis will in turn shed light on whether the rheological transition/saturation as a function of SiNP rheology modifiers is accompanied by the previously recorded conductive percolation limit [27] to expand the extrusion-based 3D printing processing capabilities of the custom AAm networks as well as expand the applications to SMART conductive gels [50]. The applications of this work fall into two major categories: (1) increasing the mechanical strength and (2) independent control of viscous and elastic modulus. First, the addition of dextran enabled us to increase the mechanical properties of the single network nanocomposites, exceeding the saturation point due to the addition of SiNPs, thus indicating an additive effect between the SiNPs and dextran. This allows for applications that require high mechanical strength such as its use as bone or cartilage scaffolds for tissue engineering [1,3]. Second, adding dextran allows researchers to tailor hydrogels with specific mechanical properties because the addition of dextran regulated increases in G’ and G” to different extents. For instance, different concentrations of dextran would allow for preparations of hydrogel nanocomposites with similar values of G’, but significantly different values of G”. These efforts will entail frequency dependent studies to confirm the correlated spectra of the elastic versus viscous modulus across custom multivariate nanocomposites. Future applications will include the development of hydrogels for drug delivery and 3D bioprinting, where tunable viscoelastic properties may increase the ease of printing and extrusion [51,52,53].

## Figures and Tables

**Table 1:** Three level factorial designs for the single network data. Values in the table are substituted for the levels on the left for the purpose of the factorial design. Each factorial design includes one Bis set (Bis Low or Bis High), one SiNP set (SiNP Low, or SiNP High), and the acrylamide set.

Level	Bis Low	Bis High	SiNP Low	SiNP High	AAm
−1	0.0625	0.125	0	3	2.5
0	0.125	0.25	1	4	5
1	0.25	0.5	2	5	10

**Table 2:** Interpenetrating network factor levels for the MANOVA analyses for investigation into the G’, G”, and tan(δ) responses.

Variable	Level (s)
Bis (x_1_)	0.0625%, 0.125%
SiNP (x_2_)	0%, 2%
Dextran MW (x_3_)	100 kDa, 500 kDa
Dextran Concentration (x_4_)	0%, 0.5%, 1%, 2%

**Table 3:** Equation (4) coefficient estimates, standard error, t ratios, and *p*-values for the three models depicted in Figure 1, with 2a corresponding to low Bis, high silica nanoparticles, 2b corresponding to high Bis, high silica nanoparticles, and 2c corresponding to low Bis, low silica nanoparticle models, respectively.

	Estimate			Std Error			t Ratio			Prob > |t|		
Term	2a	2b	2c	2a	2b	2c	2a	2b	2c	2a	2b	2c
β_0_	7300.06	7758.30	6041.55	1318.13	1137.70	125.14	5.54	6.82	48.28	<0.0001	<0.0001	<0.0001
β_1_	5964.70	5356.50	4757.83	1571.32	1356.22	149.18	3.8	3.95	31.89	0.0012	0.0009	<0.0001
β_2_	1825.21	1530.29	2010.72	1571.32	516.50	149.18	1.16	2.96	13.48	0.2598	0.0080	<0.0001
β_3_	1231.53	883.41	1189.84	610.18	526.65	96.93	2.02	1.68	12.27	0.0579	0.1098	<0.0001
β_12_	580.62	1771.09	1665.38	1873.13	615.71	177.83	0.31	2.88	9.36	0.76	0.0097	<0.0001
β_13_	73.40	280.22	599.80	727.38	627.81	115.55	0.1	0.45	5.19	0.9207	0.6604	<0.0001
β_23_	−178.42	36.75	169.53	727.38	239.09	115.55	−0.25	0.15	1.47	0.8089	0.8795	0.1587
β_123_	295.50	66.60	155.33	867.09	285.02	137.75	0.34	0.23	1.13	0.737	0.8178	0.2735

**Table 4:** ΔG’ values in units of decibel, computed from transitioning from single to interpenetrating network hydrogel nanocomposites.

Bis (%)	Dex (%)	Dex 100 kDa		Dex 500 kDa	
		SiNP 0%	SiNP 2%	SiNP 0%	SiNP 2%
0.0625	0	6.77	3.39	8.34	5.3
0.0625	0.5	4.81	2.21	6.78	3.88
0.0625	1	3.1	1.21	4.45	2.28
0.0625	2	0	0	0	0
0.125	0	4.68	4.82	5.05	5.89
0.125	0.5	4.05	3.66	4.61	5.11
0.125	1	3.23	2.52	3.81	4.05
0.125	2	2.75	1.53	2.75	1.53

## Data Availability

Data will be made available upon request to the corresponding author.

## Supplementary Materials

The following supporting information can be downloaded at: https://www.mdpi.com/article/10.3390/nano12244461/s1, Tables S1–S3: Factorial design parameters for pAAm hydrogels with low Bis and high silica nanoparticle concentrations, corresponding to Figure 2a (Table S1); Figure 2b (Table S2), and Figure 2c (Table S3); Figure S1: Elastic Moduli (G’) of pAAm hydrogel nanocomposites as a function of dextran concentration; Table S4: Linear regression parameters for the elastic moduli plots of pAAm dextran IPN hydrogel nanocomposites; Table S5: MANOVA results summarized by response in terms of *p*-values.
