# Supplementary material for: Modeling the Additive Effects of Nanoparticles and Polymers on Hydrogel Mechanical Properties Using Multifactor Analysis
Source: Nanomaterials (Basel). 2022 Dec 15;12(24):4461. doi: 10.3390/nano12244461 (PMC9785977; doi:10.3390/nano12244461)
Supplement: Supplementary file 1 [file nanomaterials-12-04461-s001.zip › nanomaterials-2080708-supplementary.pdf]

Supplementary Materials

Modeling the additive effects of nanoparticles and polymers on hydrogel mechanical properties using multifactor analysis

Emma Barrett-Catton[1], Kyle Pedersen[1], Maryam Mobed-Miremadi[1,*], and Prashanth Asuri[1,*]

Table S1. Factorial design parameters for pAAm hydrogels with low Bis and high silica nanoparticle concentrations, corresponding to Figure 2a. X_1: AAm concentration. X_2: Bis concentration. X_3: SiNP concentration.

Pattern	X_1	X_2	X_3	Y	Pred Formula Y
323	10	0.125	5	14363.3	16260.53108
123	2.5	0.125	5	2363.33	4868.822279
131	2.5	0.25	3	1770	3264.161735
122	2.5	0.125	4	2297	3552.712585
212	5	0.0625	4	8184	6648.132823
113	2.5	0.0625	5	1777	4986.943878
213	5	0.0625	5	12133.3	8132.122874
223	5	0.125	5	14363.3	8666.058546
133	2.5	0.25	5	2408	4632.579082
312	10	0.0625	4	14236	13234.63112
313	10	0.0625	5	12133.3	14422.48087
121	2.5	0.125	3	1631	2236.602891
332	10	0.25	4	17998	18514.61485
111	2.5	0.0625	3	1095	1722.823469
211	5	0.0625	3	6078	5164.142772
331	10	0.25	3	16442	17092.59819
333	10	0.25	5	18793.7	19936.63151
222	5	0.125	4	9440	7366.683622
311	10	0.0625	3	11303	12046.78138
132	2.5	0.25	4	2286	3948.370408
221	5	0.125	3	7736	6067.308699
112	2.5	0.0625	4	1608	3354.883674
231	5	0.25	3	9487	7873.640553
321	10	0.125	3	12400	13728.72032
322	10	0.125	4	14896	14994.6257
232	5	0.25	4	11983	8803.785221
233	5	0.25	5	12050	9733.92989

Table S2. Factorial design parameters for pAAm hydrogels with high Bis and high silica nanoparticle concentrations, corresponding to Figure 2b. X_1: AAm concentration. X_2: Bis concentration. X_3: SiNP concentration.

Pattern	X_1	X_2	X_3	Y	Predicted Y
322	10	0.25	4	17998	18950.11281
323	10	0.25	5	18793.7	20217.08305
232	5	0.5	4	11862	11105.89059
213	5	0.125	5	9920	8014.951814
332	10	0.5	4	28891	28304.94388
123	2.5	0.25	5	2408	3881.031179
212	5	0.125	4	9440	7229.797052
233	5	0.5	5	12236.3	11949.24178
312	10	0.125	4	14896	14272.69728
132	2.5	0.5	4	2298	2506.363945
333	10	0.5	5	29471	29847.49745
131	2.5	0.5	3	1673	2012.613946
121	2.5	0.25	3	1770	2734.340703
231	5	0.5	3	10121	10262.5394
111	2.5	0.125	3	1631	3095.204082
112	2.5	0.125	4	2297	3708.346939
321	10	0.25	3	16442	17683.14257
223	5	0.25	5	12050	9326.381802
113	2.5	0.125	5	2363.33	4321.489796
122	2.5	0.25	4	2286	3307.685941
222	5	0.25	4	11983	8521.828231
221	5	0.25	3	9487	7717.27466
331	10	0.5	3	26574	26762.39031
211	5	0.125	3	7736	6444.64229
313	10	0.125	5	14363.3	15401.87585
133	2.5	0.5	5	2332.33	3000.113944
311	10	0.125	3	12400	13143.51871

Table S3. Factorial design parameters for pAAm hydrogels with low Bis and low silica nanoparticle concentrations, corresponding to Figure 2c. X_1: AAm concentration. X_2: Bis concentration. X_3: SiNP concentration.

**Pattern**	**X_1**	**X_2**	**X3**	**Y**	**Pred Formula Y**
323	10	0.125	2	9140.67	12014.34014
123	2.5	0.125	2	1065	2482.061224
131	2.5	0.25	0	426.67	1014.304989
122	2.5	0.125	1	755	1868.918367
212	5	0.0625	1	1858	4257.415391
113	2.5	0.0625	2	530	2642.594104
213	5	0.0625	2	3284.67	5032.870748
223	5	0.125	2	4720	5659.487528
133	2.5	0.25	2	1064	2160.995465
312	10	0.0625	1	5477	8753.141299
313	10	0.0625	2	7289.33	9813.424037
121	2.5	0.125	0	413.67	1255.77551
332	10	0.25	1	12171.7	15149.2021
111	2.5	0.0625	0	47.75	1376.51077
211	5	0.0625	0	598.33	3481.960034
331	10	0.25	0	10250	13882.23186
333	10	0.25	2	14373.3	16416.17234
222	5	0.125	1	3140	4874.332767
311	10	0.0625	0	4334.67	7692.858562
132	2.5	0.25	1	769.33	1587.650227
221	5	0.125	0	1946.67	4089.178005
112	2.5	0.0625	1	283.67	2009.552437
231	5	0.25	0	3665.67	5303.613946
321	10	0.125	0	6577.33	9755.982995
322	10	0.125	1	7844	10885.16157
232	5	0.25	1	4769	6108.167518
233	5	0.25	2	6706.67	6912.721089

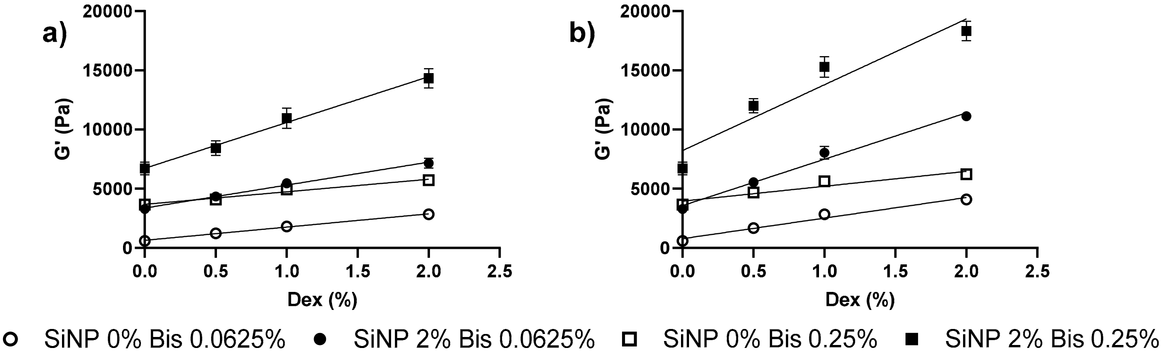

Figure S1. Elastic Moduli (G′) of pAAm hydrogel nanocomposites as a function of dextran concentration prepared using different concentrations of silica nanoparticles and Bis crosslinker, and different molecular weights of dextran (a) 100 kDa and (b) 500 kDa.

Table S4: Linear regression parameters for elastic moduli of pAAm hydrogel nanocomposites plotted against dextran concentration for samples prepared using different concentrations of silica nanoparticles and Bis crosslinker, and different molecular weights of dextran (a) 100 kDa and (b) 500 kDa.

**Hydrogel Properties**			**Linear Regression Parameters**	
**Dextran MW (kDa)**	**Bis Concentration (%)**	**SiNP Concentration (%)**	**Slope**	**R^2**
**100**	**0.0625**	**0**	1117	0.9814
		**2**	1940	0.9648
	**0.25**	**0**	1059	0.8740
		**2**	3871	0.9534
**500**	**0.0625**	**0**	1739	0.9656
		**2**	3920	0.9792
	**0.25**	**0**	1257	0.8500
		**2**	5561	0.8943

Table S5. MANOVA results summarized by response in terms of p-values.

Variable	G′	G″	Tan(δ)
**Bis concentration (x_1)**	0.00	0.06	0.00
**SiNP concentration (x_2)**	0.00	0.00	0.00
**Dex molecular weight (x_3)**	0.02	0.60	0.19
**Dex Concentration (x_4)**	0.00	0.00	0.01
